# An Efficient Fitness Function in Genetic Algorithm Classifier for Landuse Recognition on Satellite Images

**DOI:** 10.1155/2014/264512

**Published:** 2014-02-18

**Authors:** Ming-Der Yang, Yeh-Fen Yang, Tung-Ching Su, Kai-Siang Huang

**Affiliations:** ^1^Department of Civil Engineering, National Chung Hsing University, Taichung 40227, Taiwan; ^2^Department of Civil Engineering and Engineering Management, National Quemoy University, Kinmen 89250, Taiwan

## Abstract

Genetic algorithm (GA) is designed to search the optimal solution via weeding out the worse gene strings based on a fitness function. GA had demonstrated effectiveness in solving the problems of unsupervised image classification, one of the optimization problems in a large domain. Many indices or hybrid algorithms as a fitness function in a GA classifier are built to improve the classification accuracy. This paper proposes a new index, DBFCMI, by integrating two common indices, DBI and FCMI, in a GA classifier to improve the accuracy and robustness of classification. For the purpose of testing and verifying DBFCMI, well-known indices such as DBI, FCMI, and PASI are employed as well for comparison. A SPOT-5 satellite image in a partial watershed of Shihmen reservoir is adopted as the examined material for landuse classification. As a result, DBFCMI acquires higher overall accuracy and robustness than the rest indices in unsupervised classification.

## 1. Introduction

Novel techniques of image classification, including supervised and unsupervised classifications, have been developed and widely applied to the problems of pattern recognition. Supervised classification requires prior knowledge for the training of the classification model. Taking satellite image classification, for example, the prior knowledge, means the average and standard deviation of spectrum of each landuse. Such a prior knowledge has been taken as criteria and then the examined image is classified to the distinct object of interest referring to the criteria [1–5].

On the contrast, unsupervised classification can be implemented automatically by analyst-defined clustering criteria as the basis for classification rather than the training data set collected beforehand. Unsupervised classification groups a set of test data in such a way that the data within a class (cluster) are more similar in some identities to one another than in other groups. Unsupervised classification starts with a specific number of classes either arbitrarily in accordance with the research objectives or based on the analyst's expertise and then interprets all pixels within a data set into a correspondent class pixel by pixel. In accordance with such a merit, unsupervised classification is more suitable for the interpretation of environment with fragmentary land cover for areas or the image detection without prior statistics of the training data from the study field [6]. However, due to lacking of the ground truth, the accuracy of unsupervised classification is inferior to supervised classification [7]. Therefore, the accuracy improvement of unsupervised classification remains a critical issue needing a great effort.

Inspired by the nature evolution process, GA has been extensively and successfully applied to many practical problems, such as urban landscape change analysis [8], urban sprawl detection [9], multicomponent image segmentation [10], and image edge detection [11]. Therefore, how to apply GA to get better results has become a remarkable and practical topic during the past decade. GA can efficiently improve the results analyzed based upon heuristic methods away from the local solutions and then get the optimal results especially in image analysis and interpretation and artificial intelligence [12–14]. With this superiority, many GA researches were undertaken and developed.

Bandyopadhyay and Maulik [15] integrated Davies-Bouldin index, Dunn's index, Fuzzy C-means index, and C-index into GA as fitness functions for clustering analysis. Bandyopadhyay and Maulik [16] also integrated K-means into clustering GA for unsupervised clustering to improve the defect of K-means needing the initial cluster numbers a prior and getting better results. Yang and Wu [17] established partition separation index and verified its superiority by comparing it with other five noted clustering indices: partition coefficient index, partition entropy index, Fukuyama and Sugeno validity function, Xie and Beni validity function, and Davies and Bouldin validity function.

Accordingly, GA operations can start the evaluation by individuals (so-called GA strings or chromosomes) of population (initial generation) being substituted over a specified number of generations which are consisted of the strings from one initial individual that swapped some segments between two strings (so-called crossover), so as to find the optimal fitness piece by piece [18]. Particularly, instead of searching the optimal solution from a few assigned points within the searching space of the training data, GAs can initialize a group of the solution sets selected randomly and automatically from the solution space [18]. This research developed a new index by including the merits of DBI and FCMI, so-called DBFCMI, to promote the accuracy of GAs. Normally, DBI considers both the distribution of inner cluster and between clusters with membership usually defined as classical crisp logic. However, while the interaction of pixels within an image is considered, the crisp membership function of classical logic seems unsuitable for the relationship of these pixels. Instead, FCMI based on fuzzy C-means (FCM) that is extended from a method known as hard C-means is adopted in a crisp classifying application, developed by Bezdek [19], and is an extremely powerful classification method for fuzzy data. However, FCMI considers only the dispersion of inner cluster without the dispersion between clusters; therefore, the clustering efficiency could cause the beneath clustering or the excessive clustering.

In this research, the new index was verified for its feasibility and stability via various initial sets (i.e., different lengths of chromosome and numbers of populations), selection ways, and crossover ways.

## 2. Methodology

### 2.1. GA Operator

GA, based on mimicking the natural strategies of evolution, can preserve the fittest which is one of the useful optimization techniques. A genetic string, so-called an individual, is encoded of a particular solution to a problem. And the solution must be able to express characteristics of the sample space. Before an operation of GA, a number of individuals are produced for the population of initial generation. Each genetic string is usually encoded by the types of binary, integer, or real number. After the operations of crossover and mutation, the possible solutions within the solution space are obtained and calculated their fitness according to a fitness function. Repeating the operations of evolution and preserving the fittest by selection, the possible solutions could be evaluated generation by generation until the optimal solution is derived.

#### 2.1.1. GA Operating Steps

A genetic string is the foundation for establishing a genetic algorithm and could describe a possible solution to a problem. It is made of units what can represent the characters of the problem. An individual is a bit string of arbitrary units. Basically, the meaningful string length must consist of at least two and upper genes [6, 17]. In this research, the string length is set in 8 and encoded with the integer number because of the radiometric resolution of SPOT-5 image and then each unit of the gene string is comprised of 4 units because of the 4 bands of the image. The member of a generation, so-called population, also influences clustering accuracy. Besides, referring to Coley [20], Liu et al. [21], and Sivanandam and Deepa [22], the member of a generation, so-called population, 30 through 90, is adopted in this research.

Once the initial generation is randomly selected from the universal set, some strings, even number usually, with superior fitness are partially selected into the crossover pool. Afterwards, new members of population based on the operations of crossover and mutation were generated for the next generation [22, 23]. Roulette wheel selection and rank selection are the two common selection techniques that were adopted in this research.

Two typical parameters, including crossover probability (*P*_*c*_) and crossover way, must be determined. The crossover probabilities of 0.4 through 0.9 are usually suggested [20], so *P*_*c*_ of 0.8 was adopted in this research. Besides, the most common popular crossover ways, such as single-point crossover, multipoint crossover, and uniform crossover, were adopted. In addition, three other crossover forms, including three-parent crossover, ordered crossover, and shuffle crossover, were also tested.

The purpose of mutation is to prevent GA from being trapped into local optimal solutions. A low mutation probability, typically between 0.001 and 0.01, is given because a high mutation probability would change GA to random search. Sivanandam and Deepa [22] proposed that an appropriate mutation probability should be determined according to the reciprocal of string length. That is, supposing the length of the genetic string consists of 8 genes, the mutation probability is given as 1/8. Flipping mutation has been widely applied to the enlarging solution spaces [22] so to be employed in this paper.

#### 2.1.2. Fitness Function

In each generation, the priority of the genetic strings is ranked according to the fitness values calculated based on a fitness function. Through either maximizing or minimizing the fitness values generation by generation, the genetic string with the global optimum could be found to be the terminal clustering result.

Currently, many indices, such as K-means index (KMI), separation index (SI), partition separation index (PASI), Davies-Bouldin index (DBI), and fuzzy C-means index (FCMI), have been presented to be the fitness functions of GA. Among the previous indices, DBI considers the inner differences within a cluster as well as the differences among the clusters so that the better clustering results could be acquired. However, rather than considering the influence between the other clusters, the specified pixel DBI considers only the influence between the specified pixel and the cluster it belonged to. FCM basically integrated fuzzy membership function with C-means clustering and then further integrating into GA as a fitness function, so-called FCMI, can be a complementary to DBI. Therefore, in this paper, DBFCMI, integrated FCMI with DBI, is built to attempt to obtain the better clustering accuracy.

**Figure 1:**
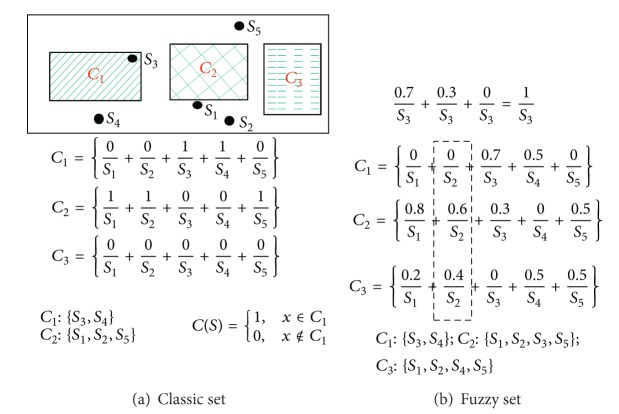
The dissimilarity between classic set and fuzzy set.

Dunn [24] developed the fuzzy C-means (FCM) which had been successfully improved and applied to the clustering analysis by Bezdek [19]. The membership function of the fuzzy C-means is used to scale the weights of a data to the clustering centers into a continuous interval [0,1] rather than the classical set with the crisp binary units 0 and 1 (see Figure 1). In Figure 1, the *C* values express the clustering centers and the *x* = {*x*_1_, *x*_2_, *x*_3_,…, *x*_*n*_} denotes an analyzed image. The subscript n means the number of the pixels of an image.

Unlike DBI, FCMI considers the influence between each pixel and all cluster centers. That is, the distance between a pixel and the pixels in the same cluster will be considerably less than the distance between a pixel and the pixels in different clusters. Of course the reciprocal influence of the former one is considered larger than the latter one. Also the membership grade is considered based on the same distance measurement. The objective function of FCMI is shown as (1), and the optimal cluster centers can be found by minimizing (1). The center of the *j*th cluster is determined by (2). Equation (3) is the membership function of *x*_*i*_ being assigned to the *j*th cluster. As for DBI, it can be obtained by the derivation of (5) [3, 15, 19]. The optimal cluster centers could be derived by maximizing DBI.

In order to demonstrate the performance of DBFCMI, this research referred to the literature of Yang and Wu [17] to apply several indices on a certain man-made data which display the different shapes of distribution. The experimental result indicates that the three indices, including Davies-Bouldin index (DBI), partition separation index (PASI), and fuzzy C-means index (FCMI), have the better performances. Therefore, this research introduced the above three indices and DBFCMI into GA for the unsupervised clustering analysis.

DBFCMI is mainly based on DBI. Furthermore, it evaluates distance between a pixel and the cluster centers based on fuzzy membership rather than the distance between the pixel and the cluster center which the pixel belonged to (see (7)). The membership function was evaluated via (8).

(a) Fuzzy C-means index (FCMI)
(1) $$\begin{array}{c} \mathrm{Min}\;\;\text{FCMI}=\sum_{j=1}^{c}\sum_{i=1}^{n}\mu_{ji}^{2}{||x_{i}-v_{j}||}^{2}, \end{array}$$
(2) $$\begin{array}{c} v_{j}=\frac{\sum_{i=1}^{n}\mu_{ji}^{2}x_{i}}{\sum_{j=1}^{n}\mu_{ji}^{2}},\;j=1,\ldots ,c,\;\;i=1,\ldots ,n, \end{array}$$
(3) $$\begin{array}{c} \mu_{ji}=\mu_{j}\left(x_{i}\right)={\left(\sum_{k=1}^{c}\frac{{||x_{i}-v_{j}||}^{2}}{{||x_{i}-v_{k}||}^{2}}\right)}^{-1}, \end{array}$$
(4) $$\begin{array}{c} \sum_{j=1}^{c}\sum_{i=1}^{n}\mu_{ji}\left(x\right)=1. \end{array}$$
(b) Davies-Bouldin index (DBI)
(5) μkn={1;||xn−vk||≤||xn−vj||,0;otherwise1≤k, j≤K; j≠k; 1≤n≤N,vk=∑n=1M(μkn)xn∑n=1M(μkn)=∑xn∈XkxnMk, 1≤k≤K,Sj=(1|Xj|∑x⊂Xj||x−vj||2)1/2,Sk=(1|Xk|∑x⊂Xk||x−vk||2)1/2,dkj,t=||vk−vj||t,Rk,t=max⁡j≠k{Sk+Sjdkj,t}, j=1,…,(K−1);  j≠k,DBi=1K∑k=1KRk,t, i=1,…,m,Max⁡  DBI=1DB.
(c) Partition separation index (PASI)
(6) $$\begin{array}{c} \text{P}{\text{S}}_{j}=\sum_{i=1}^{n}\frac{\mu_{ji}^{2}}{\mu_{M}}-\exp\left(\frac{{-\min}_{k\neq j}\left\{{||v_{j}-v_{k}||}^{2}\right\}}{\beta_{T}}\right),\\ \mu_{M}=\underset{1\leq j\leq c}{\max}\left\{\sum_{i=1}^{n}\mu_{ji}^{2}\right\},\;\;\beta_{T}=\frac{{\sum_{j=1}^{c_{j}}||v_{j}-\overset{-}{v}||}^{2}}{c_{j}},\\ \mathrm{Max}\;\;\text{PSI}\left(c\right)=\sum_{j=1}^{c}{\text{PS}}_{j}. \end{array}$$
(d) Davies-Bouldin and fuzzy C-means index (DBFCMI)
(7) $$\begin{array}{c} v_{k}=\frac{\sum_{n=1}^{M}\left(\mu_{kn}\right)x_{n}}{\sum_{n=1}^{M}\left(\mu_{kn}\right)}=\frac{\sum_{x_{n}\in X_{k}}x_{n}}{M_{k}},\;1\leq k\leq K,\\ \mu_{ji}=\mu_{j}\left(x_{i}\right)={\left(\sum_{k=1}^{c}\frac{{||x_{i}-v_{j}||}^{2}}{{||x_{i}-v_{k}||}^{2}}\right)}^{-1},\\ v_{j}=\frac{\sum_{i=1}^{n}\mu_{ji}^{2}x_{i}}{\sum_{j=1}^{n}\mu_{ji}^{2}},\;j=1,\ldots ,c,\;\;i=1,\ldots ,n, \end{array}$$
(8) $$\begin{array}{c} \sum_{j=1}^{c}\sum_{i=1}^{n}\mu_{ji}\left(x\right)=1,\\ S_{k}={\left(\frac{\sum_{j=1}^{c}\sum_{i=1}^{n}\mu_{ji}^{2}{||x_{i}-v_{i}||}^{2}}{N}\right)}^{1/2},\\ d_{kj,t}={||v_{k}-v_{j}||}_{t},\\ R_{k,t}=\underset{j,j\neq k}{\max}\{\frac{S_{k}+S_{j}}{d_{kj,t}}\},\\ {\text{DBFCM}}_{i}=\frac{1}{K}\sum_{k=1}^{K}R_{k,t},\;i=1,\ldots ,m,\\ \mathrm{Max}\;\;{\text{DBFCMI}}_{}=\frac{1}{{\text{DBFCM}}_{i}}. \end{array}$$

#### 2.1.3. Termination Criteria

There are two termination criteria for the GA operation, including the convergence of optimal solution searching or the specified number of generations that have evolved. Even though the latter termination criterion is adopted by most researchers [3, 10, 12, 21], it is too time-consuming for mega data, such as GA image classification. In this research, therefore, the convergence of optimal solution searching is set as the termination criterion for the GA operation.

### 2.2. Introduction of Study Site and SPOT-5

**Figure 2:**
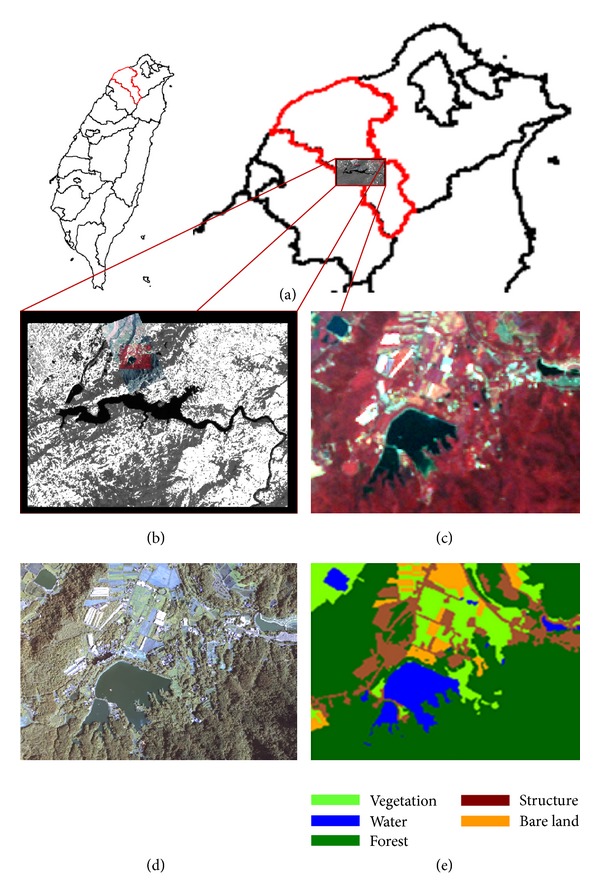
(a) Location of the Shihmen reservoir; (b) location of the study site; (c) the subset satellite image of the study site; (d) subset aerial photograph with the same studied range; (e) distributions of ground truth.

The study site is a hillside within the watershed of Shihmen reservoir located in Northern Taiwan (see Figure 2). The reservoir supplies water to 28 districts of Northern Taiwan, including 3.4 million people. The Shihmen reservoir serves a number of purposes, including irrigation, hydroelectric power, water supply, flood prevention, and sightseeing. Thus, the watershed and water monitoring of Shihmen reservoir become a very important job [25]. The economic activity of humanity in the watershed of Shihmen reservoir is an important factor that could influence the water quality. The landuse classification using remote sensing data can offer the administrator an efficient and real-time monitoring of the natural change and agricultural activities [26–28]. Landuse classification can offer the administrator an efficient and real-time monitoring for the economic activities of humanity. A SPOT-5 satellite image, which was photographed on August 19, 2006, was acquired as the experimental material. SPOT-5 satellite image has a panchromatic mode (0.48 m–0.71 m) with a spatial resolution of 5 m and a multispectral mode (0.50 m–0.59 m in green, 0.61 m–0.68 m in red, 0.78 m–0.89 m in near IR, and 1.58 m–1.75 m in short wave IR) with a spatial resolution of 10 m. The size of the subset satellite image is 181 × 171 pixels (a total of 30,951 pixels). The ground truth data was produced from an aerial photography taken on August 14, 2006. The landuse patterns include vegetation, water, forest, bare land, and structure, whose spectral centers and standard deviations are listed in Tables 1 and 2.

Most indices whenever are integrated into GA might probably cause the excessive classifying. Therefore, expect the 5 categories of landuse in accordance with the surface; the other categories determined here by GA are all defined as the 6th landuse, so-called others.

## 3. The Results

### 3.1. The Results Varying with Populations

We implemented different GA operations settings in order to verify the stable optimum of DBFCMI. In this research, the different populations consisting of 30, 60, 75, and 90 string numbers coupled with the given GA parameters, including maximal string length of 8 genes [29], roulette wheel selection, two-point crossover [24, 30], crossover probability of 0.8, and mutation probability of 0.003 [24, 31], were tested. Tables 3 and 4 show the overall accuracy and K-HAT values derived from the stability analysis varying with the different fitness indices. In the tables, DBFCMI can mostly lead to the better overall accuracy and K-HAT than the other indices, while the population is 60. In Tables 5 and 6, the best overall accuracy of 75.5% and the best K-HAT of 0.48 were derived, while the population is assigned to 30 based on DBFCMI.

**Figure 3:**
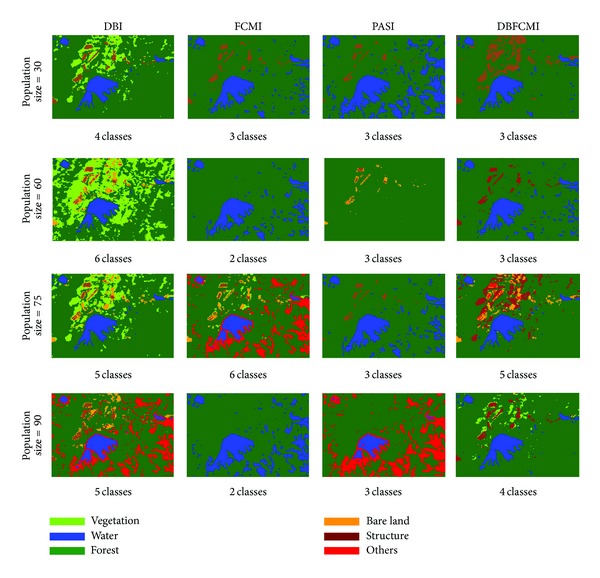
The best result interpreted by the different indices varying with populations.

The image classification results corresponding to overall accuracy and K-HAT values in Tables 3 and 4 are shown in Figure 3. Figure 3 shows that there were only 3 classes detected based on the best overall accuracy and K-HAT of 75.4% and 0.48%. It is remarkable that DBI mostly can get the number of classifications as many as the ground truth. However, the pixels would mostly be assigned in incorrect classes especially in vegetation. Unlike DBFCMI, although the numbers of classifications are mostly under the number of real landuses, nevertheless, the distribution of each landuse is more corresponding to the ground truth. In Figure 3, it is remarkable that DBFCMI can distinguish structure from the other landuses more accurately and the distribution of structure is more corresponding to ground truth than the other associated models.

### 3.2. The Results Varying with Selection Ways

Optimal solution by elite selection in GA operations includes many ways. Two of them are adopted widely, that is, roulette wheel selection and rank selection. Thus, the two selection ways applied to the four indices were evaluated based on overall accuracy and K-HAT as well. The testing results presented in Tables 5 and 6 demonstrate that the DBFCMI can get higher values in both selection ways and roulette wheel selection outperforms rank selection for most indices except PASI.

**Figure 4:**
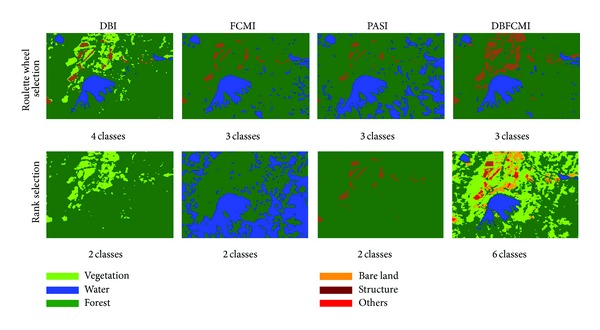
The best result interpreted by the different indices varying with selection ways.

Figure 4 shows the classified results varying with selection ways related to different indices. Comparing the same six classes between DBI (see Figure 3; population size is equal to 60) and DBFCMI, DBFCMI can distinguish more pixels of bare land than DBI properly and thus can get higher values of both overall accuracy and K-HAT. The results are used once again to verify the accuracy and stability of DBFCMI.

### 3.3. The Results Varying with Crossover Ways

In the way of crossover approaches, including single-point crossover (P1), two-point crossover (P2), multipoint crossover (P3), three-parent crossover (P4), ordered crossover (P5), and shuffle crossover (P6), related to the different indices, they were also tested in this research (see Tables 7 and 8) for the accuracy and stability analysis. Obviously, the better overall accuracy and K-HAT values could be derived from DBFCMI than the other indices as well as the advanced GA operations.

**Figure 5:**
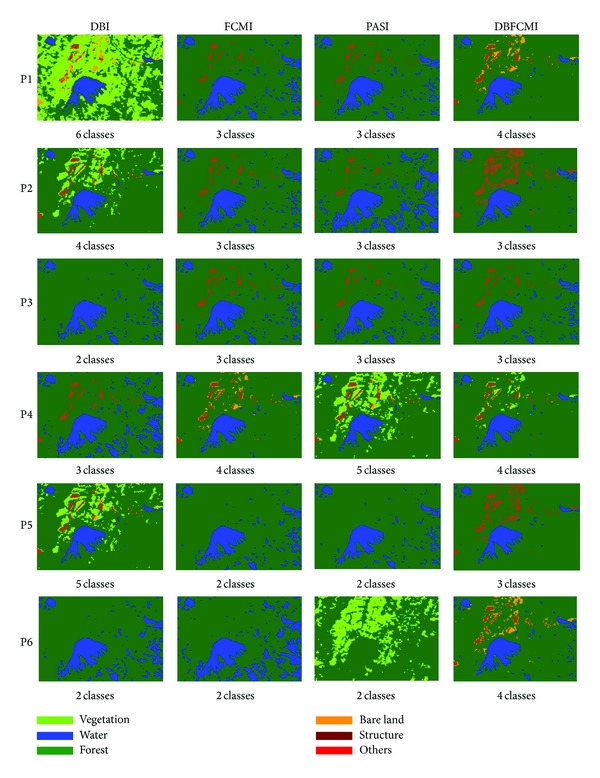
The best result interpreted by the different indices varying with crossover ways.

**Figure 6:**
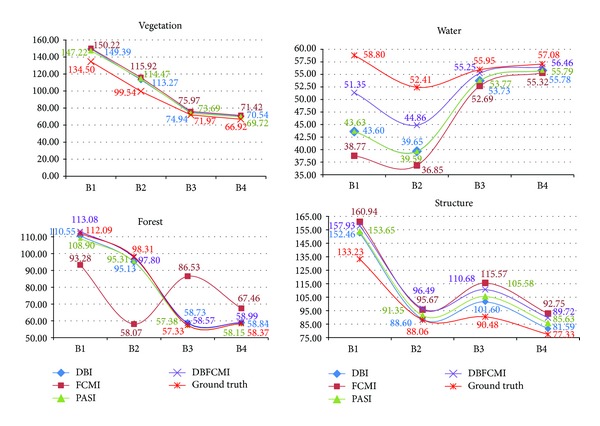
Curve comparison of spectral centers between ground truth and landuse classified based on optimal solution of four indices.

Figure 5 is the classified images related to Tables 7 and 8. Among these results from the indices varying with the different crossover ways, DBFCMI still can get the higher values of overall accuracy and K-HAT in substance except a K-HAT value of FCMI with the multipoint crossover way (see Table 8). Also, DBFCMI presents its power of distinguishing structure from the other landuses again although the classes are mostly beneath the number of ground truth. Besides, according to Figures 5 and 6, it is presented that while DBI is integrated with the single-point crossover way (P1) and two-point crossover way (P2), the results will have the higher potential in excessive classifying than the other indices.

## 4. Results Analysis

Figure 6 shows the spectral centrals of the optimal solutions based on the four indices varying with different GA operations versus ground truth. In the figure, the difference of spectral centers between the optimal solutions and ground truth can be observed evidently. Among the five landuses, bare land cannot be determined by all of the four indices with their optimal models. Even though bare land can be distinguished from the other landuses with some of the GA operations, the distribution of it principally disagrees with ground truth. On the contrary, among the optimal solutions, the centers of forest can be determined much closer to the ground truth in both classified centers and distribution than the other landuses due to its wide spectrum variation, and then vegetation, structure, and water are in sequence. DBFCMI can only determine three kinds of landuse (i.e., forest, water, and structure), however the spectral centers of the three landuses classified by DBFCMI seem to be closer to the ground truth than the other indices especially in forest and water. Among the GA classifications with four indices, FCMI results in the greatest difference between the classified centers and ground truth especially in forest for all bands. It can be observed in Figure 6 that DBFCMI presents its superiority of conformation in the distribution. On the other hand, the results of DBI and PASI can get the optimal solution inferior to DBFCMI; nevertheless, comparing to FCMI, their classified centers are not only closer to ground truth but also with a curve as smooth as the ground truth is.

According to the foregoing analysis, it is worth to notice that rather than classifying the number of landuses accurately but inconformity with the distribution, the ability of distribution determination possesses the crucial influence upon the optimal solution.

**Figure 7:**
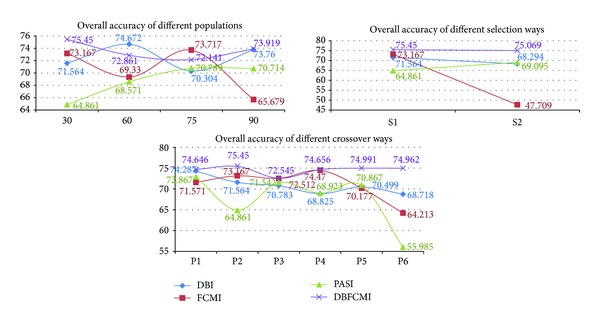
Curve comparison of overall accuracy based on four indices varying with different GA operations.

**Figure 8:**
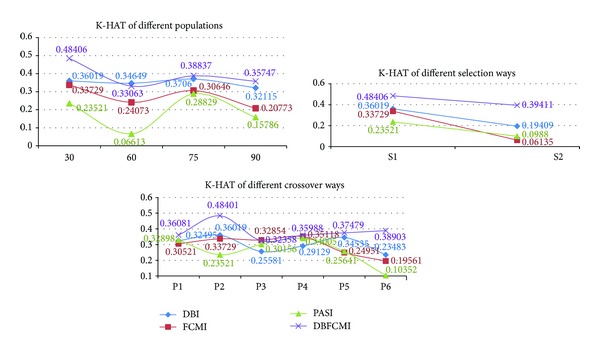
Curve comparison of K-HAT based on four indices varying with different GA operation.

Figures 7 and 8 are the curve comparison of overall accuracy and K-HAT based on four indices varying with the different GA operations, respectively. It can be observed the higher values and stability of overall accuracy and K-HAT are presented, while DBFCMI is adopted and then DBI, FCMI, and PASI are in sequence. FCMI and PASI are based on the fuzzy theory and the curves of their accuracy related to the different GA operations vibrate violently rather than a curve as smooth as DBFCMI and DBI. It is remarkable that the overall accuracy and K-HAT of PASI are inferior to the other indices mostly in our test.

**Figure 9:**
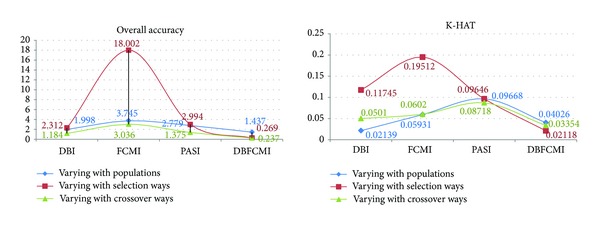
Standard deviation comparisons of overall accuracy and K-HAT between the four indices varying with different GA operations.

Figure 9 is the standard deviation of overall accuracy and K-HAT. It presents the comparison curves of four indices varying with the different GA operations. In the figure, the smallest difference between the different GA operations is presented still at the index of DBFCMI in both overall accuracy and K-HAT. And then DBI, PASI, and FCMI are in sequence. Although four indices are varying with the different selections, FCMI has the largest difference between the four indices varying with the other GA operations. This means that the unstable analyzed results are presented at FCMI more possible than the other indices.

## 5. Conclusion

This paper presented a novel fitness index, DBFCMI, in GA process for the unsupervised classification of SPOT-5 satellite image. For comparison, three indices, including Davies-Bouldin index (DBI), fuzzy C-means index (FCMI), and partition separation index (PASI), were also adopted in GA classification. The conclusion is drawn as follows.

- Spectra of bare land and vegetation are as similar as forest in the tested image, so that it is difficult to discriminate the three landuses from each other with GA classifier. Therefore, in most conditions the best associated model of GA can only distinguish bare land and vegetation into forest.
- Overall accuracy and K-HAT are stronger related to distribution of classified landuse than the number of classifications. Besides, except distribution, another critical influence is depending upon the area of landuse especially the landuse with a large area. The best overall accuracy of 75.5% and the best K-HAT of 0.48 were acquired by DBFCMI, with merely three landuses, including forest, water, and structure. However, except the distribution of water and structure which can be determined more identical than the other indices, the largest region of forest can be determined appropriately by DBFCMI as well. Therefore, the influence is not so critical even though the spectra of bare land and vegetation are too similar to forest to be distinguished.
- Comparing with the three indices including DBI, FCMI, and PASI, FCMI and PASI are both based on fuzzy theory so that all the other cluster centers will be considered to influence each independent pixel more or less according to the distance between the pixel and the centers. On the contrary, DBI index based on the classic set theory identifies each pixel in the training data into only one cluster that reduces computation time but results in moderate accuracy. Basically, the physical phenomenon of the spectrum reflection resulted from the neighborhood objects is inevitable. However, sometimes the ideal performances of image classification are obtained by GA coupled with DBI rather than FCMI or PASI. DBFCMI has possessed both advantages of DBI and fuzzy theory and all the examination of this research had been demonstrated that it is effective in the unsupervised image classification. As a result, the best overall accuracy of DBFCMI, DBI, FCMI, and PASI is 75.5%, 75.0%, 74.9%, and 74.2% separately. DBFCMI presents 0.75% increment in the average of the other indices. Overall accuracy is promoted about 1.01% in average. On the other hand, the best K-HAT of DBFCMI, DBI, FCMI, and PASI is 0.48, 0.37, 0.39, and 0.39 separately. DBFCMI presents 0.1 increments in the average of the other indices. Accordingly, DBFCMI can almost promote K-HAT value to 26.13% in average.

## Figures and Tables

**Table 1:** Spectral centers of landuse.

Landuse	Band			
	Spectral center			
	B1 (NIR)	B2 (G)	B3 (R)	B4 (SWIR)
Vegetation	134.5	99.5	72.0	66.9
Water	58.8	52.4	55.9	57.1
Forest	112.1	98.3	57.3	58.4
Bare land	128.9	93.9	72.9	65.9
Structure	133.2	88.1	90.5	77.3

**Table 2:** Standard deviations of the spectrum.

Landuse	Band					
	Standard deviation				Overall standard deviation	Threshold (2*σ*)
	B1	B2	B3	B4		
Vegetation	27.2	22.1	7.2	4.0	27.2	22.1
Water	13.5	12.8	11.5	6.6	13.5	12.8
Forest	13.4	11.2	7.4	4.2	13.4	11.2
Bare land	17.7	14.4	12.4	5.5	17.7	14.4
Structure	27.3	16.6	32.3	22.1	27.3	16.6

**Table 3:** Overall accuracy of each index varying with populations.

Population	Index				
	Overall accuracy (%)				
	DBI	FCMI	PASI	DBFCMI	Average
30	71.6	73.2	64.9	75.5	71.3
60	74.7	69.3	68.6	72.9	71.4
75	70.3	73.7	70.8	72.1	71.7
90	73.8	65.7	70.7	73.9	71.0
Standard deviation	2.0	3.7	2.8	1.4	—

*String length: 8, selection way: roulette wheel selection, crossover rate: 0.8, crossover way: two-point crossover, and mutation rate: 0.003.

**Table 4:** K-HAT of each index varying with populations.

Population	Index				
	K-HAT				
	DBI	FCMI	PASI	DBFCMI	Average
30	0.36	0.34	0.24	0.48	0.34
60	0.35	0.24	0.07	0.33	0.25
75	0.37	0.31	0.29	0.39	0.34
90	0.32	0.21	0.16	0.36	0.26
Standard deviation	0.02	0.06	0.10	0.04	—

*String length: 8, selection way: roulette wheel selection, crossover rate: 0.8, crossover way: two-point crossover, and mutation rate: 0.003.

**Table 5:** Overall accuracy of each index varying with selection ways.

Selection way	Index				
	Overall accuracy (%)				
	DBI	FCMI	PASI	DBFCMI	Average
Roulette wheel selection	71.6	73.2	64.9	75.5	71.3
Rank selection	68.3	47.7	69.1	75.1	65.0
Standard deviation	2.3	18.0	3.0	0.3	—

*String length: 8, population: 30, crossover rate: 0.8, crossover way: two-point crossover, and mutation rate: 0.003.

**Table 6:** K-HAT of each index varying with selection ways.

Selection way	Index				
	K-HAT				
	DBI	FCMI	PASI	DBFCMI	Average
Roulette wheel Selection	0.36	0.34	0.24	0.48	0.34
Rank selection	0.19	0.06	0.10	0.39	0.19
Standard deviation	0.12	0.20	0.10	0.02	—

*String length: 8, population: 30, crossover rate: 0.8, crossover way: two-point crossover, mutation rate: 0.003.

**Table 7:** Overall accuracy value of each model varying with crossover ways.

Crossover way	Index				
	Overall accuracy (%)				
	DBI	FCMI	PASI	DBFCMI	Average
P1	74.3	71.6	72.9	74.6	73.3
P2	71.6	73.2	64.9	75.5	71.3
P3	70.8	72.5	71.5	72.5	71.8
P4	68.8	74.5	68.9	74.7	71.7
P5	70.5	70.2	70.9	75.0	71.6
P6	68.7	64.2	56.0	75.0	73.3
Standard deviation	1.2	3.0	1.4	0.2	—

*String length: 8, population: 30, selection way: roulette wheel selection, crossover rate: 0.8, and mutation rate: 0.003.

**Table 8:** K-HAT value of each model varying with crossover ways.

Crossover way	Index				
	K-HAT				
	DBI	FCMI	PASI	DBFCMI	Average
P1	0.32	0.31	0.33	0.36	0.33
P2	0.36	0.34	0.24	0.48	0.34
P3	0.26	0.33	0.30	0.32	0.30
P4	0.29	0.35	0.34	0.36	0.34
P5	0.35	0.25	0.26	0.37	0.31
P6	0.23	0.20	0.10	0.39	0.23
Standard deviation	0.05	0.06	0.09	0.03	—

*String length: 8, population: 30, selection way: roulette wheel selection, crossover rate: 0.8, and mutation rate: 0.003.

## Abbreviations

*x*_*n*_: Pixel *n* with grey values *x*
*n*: Total number of pixels
*K*: Total number of clusters
*k*_*n*_: The membership value of *n*th pixel (if the pixel *n* belongs to *k*, *k*_*n*_ = 1; otherwise, *k*_*n*_ = 1)
*v*_*k*_: The centroid of *k*th cluster
*M*_*k*_: The number of pixels belonging to the *k*th cluster
*S*_*k*_: Standard deviation of the pixels in the *k*th cluster
*d*_*kj*,*t*_: Minkowski distance of order *t* between the *k*th and *j*th centroids. (here, number 2 is chosen for *t*)
*i*: The *i*th chromosome
*m*: Total chromosomes of each generation
*μ*_*ji*_: Membership function of pixel *x*_*i*_ belonging to the *j*th cluster
*c*_*j*_: Total pixels of *j*th cluster
$\overset{-}{v}$: The mean of all of the cluster centers.

## References

[1] Avery TE, Berlin GL (1992). *Fundamentals of Remote Sensing and Airphoto Interpretation*.

[2] Su TC, Yang MD, Wu TC, Lin JY (2011). Morphological segmentation based on edge detection for sewer pipe defects on CCTV images. *Expert Systems with Applications*.

[3] Yang MD (2007). A genetic algorithm (GA) based automated classifier for remote sensing imagery. *Canadian Journal of Remote Sensing*.

[4] Yang MD, Su TC (2009). Segmenting ideal morphologies of sewer pipe defects on CCTV images for automated diagnosis. *Expert Systems with Applications*.

[5] Yang MD, Su TC (2008). Automated diagnosis of sewer pipe defects based on machine learning approaches. *Expert Systems with Applications*.

[6] Yang MD, Yang YF, Hsu SC (2004). Application of remotely sensed data to the assessment of terrain factors affecting the Tsao-Ling landslide. *Canadian Journal of Remote Sensing*.

[7] Lillesand TM, Kiefer RW, Chipman JW (2007). *Remote Sensing and Image Interpretation*.

[8] Tang J, Wang L, Yao Z (2007). Spatio-temporal urban landscape change analysis using the Markov chain model and a modified genetic algorithm. *International Journal of Remote Sensing*.

[9] Tong X, Zhang X, Liu M (2010). Detection of urban sprawl using a genetic algorithm-evolved artificial neural network classification in remote sensing: a case study in Jiading and Putuo districts of Shanghai, China. *International Journal of Remote Sensing*.

[10] Awad MM, Chehdi K (2009). Satellite image segmentation using hybrid variable genetic algorithm. *International Journal of Imaging Systems and Technology*.

[11] Jubai A, Jing B, Yang J (2006). Combining fuzzy theory and a genetic algorithm for satellite image edge detection. *International Journal of Remote Sensing*.

[12] Zhang CJ, Wang XD (2009). Typhoon cloud image enhancement and reducing speckle with genetic algorithm in stationary wavelet domain. *IET Image Processing*.

[13] Yang MD, Su TC (2007). An optimization model of sewage rehabilitation. *Journal of the Chinese Institute of Engineers*.

[14] Yang MD, Su TC (2006). Automation model of sewerage rehabilitation planning. *Water Science and Technology*.

[15] Bandyopadhyay S, Maulik U (2002). Genetic clustering for automatic evolution of clusters and application to image classification. *Pattern Recognition*.

[16] Bandyopadhyay S, Maulik U (2002). An evolutionary technique based on K-Means algorithm for optimal clustering in R^N^. *Information Sciences*.

[17] Yang MS, Wu KL A new validity index for fuzzy clustering.

[18] Bazi Y, Melgani F, Bruzzone L, Vernazza G (2009). A genetic expectation-maximization method for unsupervised change detection in multitemporal SAR imagery. *International Journal of Remote Sensing*.

[19] Bezdek JC (1981). *Pattern Recognition with Fuzzy Objective Function Algorithms*.

[20] Coley DA (1999). *An Introduction to Genetic Algorithms for Scientists and Engineers*.

[21] Liu Z, Liu A, Wang C, Niu Z (2004). Evolving neural network using real coded genetic algorithm (GA) for multispectral image classification. *Future Generation Computer Systems*.

[22] Sivanandam SN, Deepa SN (2008). *Introduction to Genetic Algorithms*.

[23] Maiti AK, Maiti M (2008). Discounted multi-item inventory model via genetic algorithm with Roulette wheel selection, arithmetic crossover and uniform mutation in constraints bounded domains. *International Journal of Computer Mathematics*.

[24] Dunn JC (1974). A fuzzy relative of the ISODATA process and its in detecting compact well-separated clusters. *Journal of Cybernetics*.

[25] Kuo JT, Lung WS, Yang CP, Liu WC, Yang MD, Tang TS (2006). Eutrophication modelling of reservoirs in Taiwan. *Environmental Modelling and Software*.

[26] Yang MD, Lin JY, Yao CY, Chen JY, Su T-C, Jan CD (2011). Landslide-induced levee failure by high concentrated sediment flow—a case of Shan-An levee at Chenyulan River, Taiwan. *Engineering Geology*.

[27] Lin JY, Yang MD, Lin BR, Lin PS (2011). Risk assessment of debris flows in Songhe Stream, Taiwan. *Engineering Geology*.

[28] Yang MD, Su TC, Hsu CH, Chang KC, Wu AM (2007). Mapping of the 26 December 2004 tsunami disaster by using FORMOSAT-2 images. *International Journal of Remote Sensing*.

[29] Yang YF, Lohmann P, Heipke C Genetic algorithms for the unsupervised classification of satellite images.

[30] Chen CC, Lin CS (2007). A GA-based nearly optimal image authentication approach. *International Journal of Innovative Computing, Information and Control*.

[31] Kim JB, Kim HJ (2003). GA-based image restoration by isophote constraint optimization. *EURASIP Journal on Advances in Signal Processing*.

